# Nascent mRNA damage: depot and disposal

**DOI:** 10.1038/s41392-024-01900-6

**Published:** 2024-08-09

**Authors:** Mark Helm, Marie-Luise Winz

**Affiliations:** Institute of Pharmaceutical and Biomedical Science (IPBS), Johannes Gutenberg University Mainz, Staudingerweg 5, 55128 Mainz, Germany

**Keywords:** Senescence, RNA splicing

In a recent study published in *Cell*, Zhou et al.^1^ have characterized a highly unusual behavior of specialized stress granules (SG), driven by metabolism of UV-damaged pre-mRNA. These cytoplasmic SGs are characterized by an unusual content of intronic sequences and the double-stranded RNA (dsRNA) specific helicase DHX9, both of which are usually found in the nucleus.

**Fig. 1:**
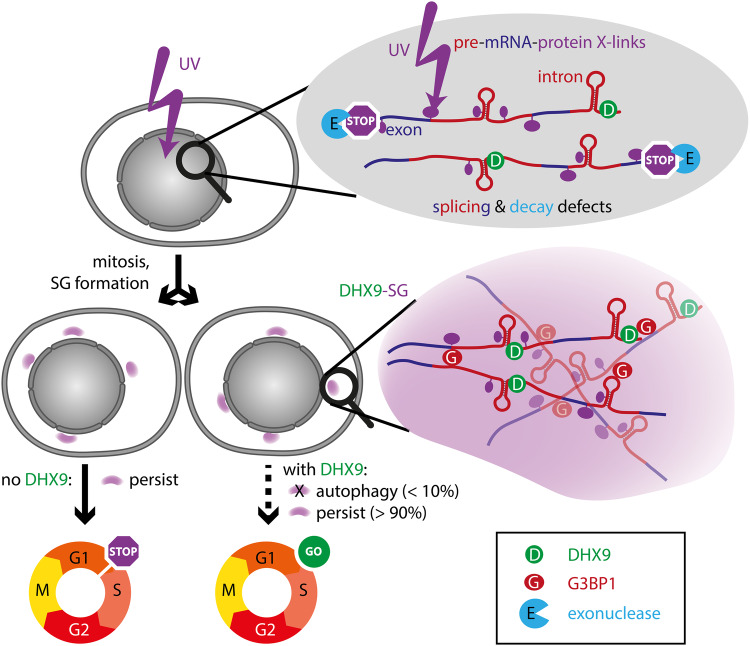
Schematic overview of key findings. UV irradiation induces crosslinks between pre-mRNA and nuclear proteins, leading to splicing and 3’-end formation defects, with enhanced intron retention. Decay of these aberrant, lesioned RNAs, e.g., by exonucleases (light blue, “E”), fails. dsRNA-targeted helicase DHX9 (green, “D”) recognizes such damage. Upon mitosis (or leakiness of the nuclear envelope), DHX9-SGs form in G1 phase daughter cells. SG formation is promoted by the canonical SG-nucleating protein G3BP1 (red, “G”). It is likely that RNA and proteins form a 3D network, as known from other types of published SGs. Cells can only resume cell cycle if SGs are cleared by autophagy, dependent on the presence of DHX9

SGs are membraneless organelles often appearing in the context of cellular stress. Certain types of SGs, such as those induced by heat shock, osmotic stress, or various toxic compounds, contain mature mRNAs in different stages of translation-readiness, and are associated with, but not required for, translation shutdown. Initially, the Akhtar lab observed relocation of the helicase DHX9 from the nucleus to cytosolic SGs upon UV-irradiation. Formation of these specific SGs depended specifically on UV-irradiation, but not on other nucleic acid damaging stresses. While UV-dependent SGs had previously been described,^2^ this team could demonstrate the dependence on RNA crosslinking reactions by leveraging the photoreactivity of s4U, a modified ribonucleoside used to make nascent RNA susceptible to crosslinking by long-wave UV light, which does not otherwise cause nucleic acid crosslinking. In addition, the group accomplished the impressive feat of developing a particle sorting technique for membraneless organelles—termed FANCI (fluorescence-activated non-membrane condensates isolation)—for the purification of SGs. RNAseq analysis of DHX9-SGs showed a striking enrichment of introns, in contrast to SGs induced by other agents. Further sequence-analyses pointed to an increase in pre-mRNA processing errors. In addition to retention of intronic sequences, this included aberrant 3’ end formation. While the latter might in part derive from the use of (non-physiological) s4U,^3^ the authors provide further evidence that significant amounts of DHX9-SG-RNAs consist of Pol II-dependent, nascent mRNA, rather than mature RNA species (Fig. 1).

At this point, the intriguing question imposed itself, how intronic sequences, presumably crosslinked in the nucleus, might relocate into cytosolic SGs in the absence of any known transporter for such structures. Here, the observation that DHX9-SGs, only formed after cell division led to the idea that the removal of the nuclear envelope during mitosis might enable relocation. This was supported by chemically induced nuclear envelope leakiness, upon which DHX9-SGs formed without prior mitosis. As is the case for canonical SGs, formation of DHX9-SGs is promoted by the canonical SG-nucleating protein G3BP1. However, DHX9-SGs in daughter cells descending from the UV-irradiated parental cell, themselves caused translation shutdown through PKR-mediated phosphorylation of eIF2α. This is a feature known from RNA-dependent innate immune responses, which clearly sets DHX9-SGs apart from other, canonical SGs. Another known molecular stressor, dsRNA, is contained in the SGs, but reduced in the presence of the helicase DHX9. Only a small fraction of daughter cells appeared to successfully resolve the DHX9-SGs through autophagy. In accordance with prior reports,^2^ this would be a prerequisite for further cell divisions.

The paper features not just a single, but a plethora of discoveries, linking an enigmatic type of SG to a new RNA damage detoxification pathway. The authors convincingly demonstrate not only the unique nature of DHX9-SGs but characterize the stress response revolving around them at the molecular level. As an enabling methodology, the fancy-sounding (pun intended) sorting approach finally allowed analyses of their molecular composition. A crucial difference to canonical SGs was found at the RNA level: While the latter comprise mature and therefore polyadenylated mRNA, the authors showed for the first time that UV-SGs did contain RNA at all, and that it was enriched in protein-crosslinked, intronic RNA sequences, indicating DHX9-SGs as depots for the segregation of damaged pre-mRNA. Their removal by autophagy constitutes a whole new RNA degradation pathway, as a backup mechanism when canonical, nuclear degradation fails due to crosslinking damage. A pathway for disposal of UV-crosslinked, mature mRNA was only recently described to involve ribosome collisions,^4^ implicating translation. However, damaged pre-mRNA, now discovered outside the nucleus, is not translated and therefore cannot enter this pathway. The Akthar lab has now filled this gap, by answering an important question not previously asked, namely: “where does damaged nascent mRNA get degraded?”.

In so opening a new area of the RNA damage field, numerous new and exciting questions come to mind at the molecular as well as at the cellular level.

As an example, DHX9 is the defining molecular entity of this pathway, but details of the crucial recognition event remain open to speculation. The authors invoke RNAseq data that point to RNA-protein crosslinked structures. DHX9, as a helicase encountering such a structure, could plausibly stall, possibly undergoing a conformational change. Such a structure, characteristic of a UV event, might then constitute a danger signal that eventually triggers the observed formation of SGs after mitosis.

The Akthar lab has demonstrated that UV-crosslinking triggers formation of DHX9-SGs not only in keratinocytes and human epidermal equivalent organoids, thus in cells typically exposed to sunlight, hence UV, but also in HeLa cells. Descending from human cervical carcinoma epithelial cells, those represent a cell type not physiologically subjected to UV-irradiation. Although HeLa cells are notorious for their divergence from physiological properties due to their cancerous provenance and long-time culturing, this implies that DHX9-SGs are neither confined to UV-exposed cells, nor UV as the only trigger. Interestingly, the same mechanism that targets mature UV-crosslinked RNA also acts on chemically crosslinked RNA and has been suggested as a surveillance pathway for aldehyde stress.^5^ Similarly, DHX9-SGs may also form under aldehyde, and potentially even other crosslink-inducing stresses. Moreover, as shown in the present paper, DHX9-SGs can form (though less efficiently) upon splicing inhibition, suggesting this pathway could serve as a general response to different splicing and possibly pre-mRNA processing stresses. This makes DHX9, which is ubiquitously expressed, a potentially crucial surveillance protein, whose importance in the context of skin as opposed to organoids, as well as non-cancerous, non-UV-exposed cell types remains to be explored.
